# *N*′-[(*E*)-3-Bromo-5-chloro-2-hy­droxy­benzyl­idene]furan-2-carbohydrazide

**DOI:** 10.1107/S160053681401085X

**Published:** 2014-05-17

**Authors:** A. Sundar, S. Ranjith, G. Rajagopal

**Affiliations:** a Department of Chemistry, Madras Medical College, Chennai 600 003, India; b Department of Physics, SRM University, Ramapuram Campus, Chennai 600 005, India

## Abstract

In the title compound, C_12_H_8_BrClN_2_O_3_, the furan ring makes a dihedral angle of 17.2 (2)° with the six-membered ring. An intra­molecular O–H⋯N hydrogen bond stabilizes the mol­ecular conformation. In the crystal, N–H⋯O hydrogen bonds connect the mol­ecules into chains running along the *c*-axis direction. The crystal packing is additionally stabilized by C—H⋯O inter­actions into a three-dimensional supramolecular architecture.

## Related literature   

Heterocyclic carbohydrazides form stable metal chelates which find applications in mol­ecular sensing, see: Bakir & Brown (2002 ▶). For the biological activity of hydrazones derived from isoniazid (systematic name: isonicotinohydrazide), see: Rollas & Kucukguzel (2007 ▶). For related structures, see: Prabhu *et al.* (2011 ▶); Bikas *et al.* (2010 ▶); Prasanna *et al.* (2013 ▶).
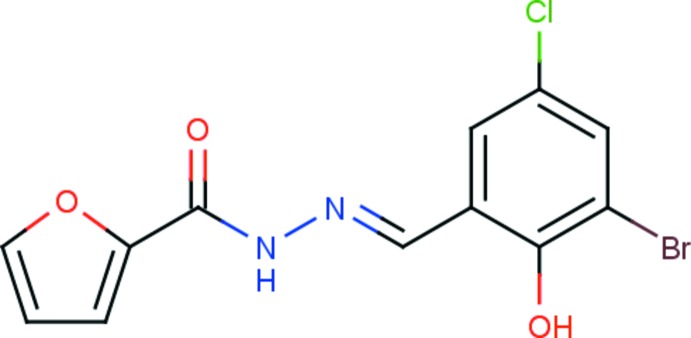


## Experimental   

### 

#### Crystal data  

C_12_H_8_BrClN_2_O_3_
*M* *_r_* = 343.56
Monoclinic, 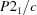

*a* = 16.7237 (9) Å
*b* = 7.7455 (4) Å
*c* = 10.1868 (5) Å
β = 93.557 (2)°
*V* = 1316.99 (12) Å^3^
*Z* = 4
Mo *K*α radiation
μ = 3.33 mm^−1^
*T* = 293 K
0.35 × 0.30 × 0.25 mm

#### Data collection  

Bruker AAPEXII CCD Diffractometer
Absorption correction: multi-scan (*SADABS*; Bruker, 2004 ▶) *T*_min_ = 0.324, *T*_max_ = 0.435
13728 measured reflections
2996 independent reflections
2029 reflections with *I* > 2σ(*I*)
*R*_int_ = 0.032

#### Refinement  

*R*[*F* ^2^ > 2σ(*F* ^2^)] = 0.034
*wR*(*F* ^2^) = 0.085
*S* = 1.02
2996 reflections
172 parameters
H-atom parameters constrained
Δρ_max_ = 0.39 e Å^−3^
Δρ_min_ = −0.34 e Å^−3^

### 

Data collection: *APEX2* (Bruker, 2004 ▶); cell refinement: *APEX2* and *SAINT* (Bruker, 2004 ▶); data reduction: *SAINT* and *XPREP* (Bruker, 2004 ▶); program(s) used to solve structure: *SHELXS97* (Sheldrick, 2008 ▶); program(s) used to refine structure: *SHELXL97* (Sheldrick, 2008 ▶); molecular graphics: *ORTEP-3 for Windows* (Farrugia, 2012 ▶); software used to prepare material for publication: *PLATON* (Spek, 2009 ▶).

## Supplementary Material

Crystal structure: contains datablock(s) global, I. DOI: 10.1107/S160053681401085X/bt6980sup1.cif

Structure factors: contains datablock(s) I. DOI: 10.1107/S160053681401085X/bt6980Isup2.hkl

Supporting information file. DOI: 10.1107/S160053681401085X/bt6980Isup3.cml

CCDC reference: 1002445

Additional supporting information:  crystallographic information; 3D view; checkCIF report

## Figures and Tables

**Table 1:** Hydrogen-bond geometry (Å, °)

*D*—H⋯*A*	*D*—H	H⋯*A*	*D*⋯*A*	*D*—H⋯*A*
N1—H1*A*⋯O2^i^	0.86	2.14	2.953 (2)	157
C3—H3⋯O1^ii^	0.93	2.44	3.324 (3)	159
C6—H6⋯O2^i^	0.93	2.50	3.263 (3)	139
O3—H3*A*⋯N2	0.82	1.84	2.564 (3)	146

Symmetry codes: (i) 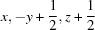; (ii) 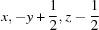.

**Fig. 1.**
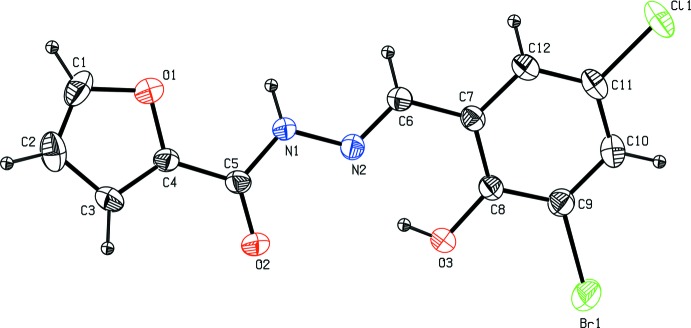
The molecular structure of the title compound, showing the atomic numbering and displacement ellipsoids drawn at 30% probability level.

**Fig. 2.**
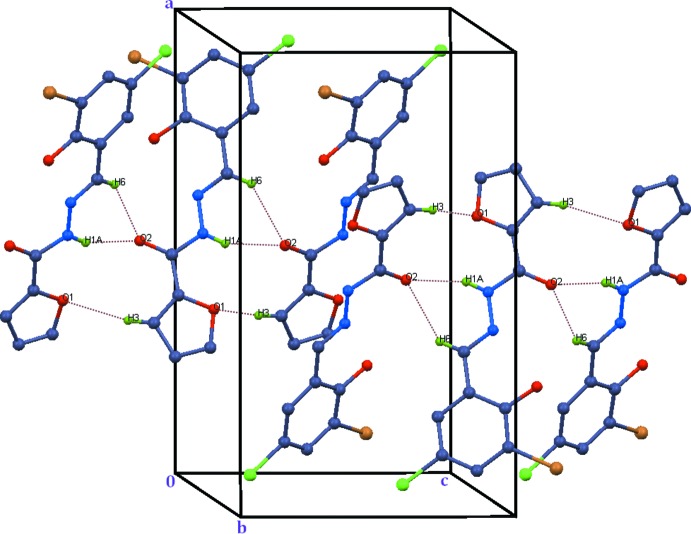
The crystal structure showing the R22(10) motif and also the formation of bifurcated R12(6) ring motif. For the sake of clarity, the H atoms not involved in the motif have been omitted.

## supplementary crystallographic information

### 1. Comment

Heterocyclic carbohydrazides are compounds with a wide spectrum of biological and analytical applications. They form stable metal chelates which find applications in molecular sensing (Bakir & Brown, 2002). A number of hydrazones derived from isoniazid were reported to be active antitubercular agents and were found to be less toxic than isoniazid (Rollas & Kucukguzel, 2007). Against this background, and in order to obtain detailed information on the molecular conformation in the solid state, an X-ray study of the title compound was carried out.

The X-ray analysis confirms the molecular structure and atom connectivity as illustrated in Fig. 1. The molecule exists in a *E* configuration with respect to the C6=N2 bond, with the C7—C6—N2—N1 torsion angle of 179.1 (2)°. The bond lengths and angles in the carbohydrazide group of the title compound can be compared with the related structures (Prabhu *et al.*, 2011; Bikas *et al.*, 2010). The furan ring makes a dihedral angle of 17.2 (2)° with the six-membered ring. The N2—N1—C5—O2 torsion angle of -0.1 (4)° indicates the *cis* configuration of the O2 atom with respect to the hydrazine nitrogen atom N2. The bond distances C6═N2 [1.275 (3) Å] and C5═O2 [1.224 (3) Å] are very close to the formal double C═N and C═O bond lengths (Prasanna, *et al.*, 2013) confirming that the carbohydrazide exists in solid state as an amido tautomer. An intramolecular O–H···N hydrogen bond stabilized the molecular conformation. Intermolecular N–H···O hydrogen bonds connect the molecules to chains running along the *c* axis. The crystal packing is further stabilized by C–H···O hydrogen bonds.

### 2. Experimental

*N*'-[(*E*)-(3-bromo-5-chloro-2-hydroxyphenyl)methylidene]furan-2-carbohydrazide, ligand was synthesized by Schiff-base condensation furan-2-carbohydrazide and 3-bromo-5-chlorosalicylaldehyde as shown in Scheme-1. 3-bromo-5-chloro salicylaldehyde (3.0 mmol) in methanol (0.75 g) was stirred in a round bottom flask followed by drop wise addition of methanolic solution of furan-2-carbohydrazide (3.0 mmol). The reaction mixture was stirred for 3 h. The resulting white solid was removed by filtration and washed with cold ethanol and dried in vacuum over anhydrous CaCl_2_·*M*.p:180°C, yield: 80%. Single crystals suitable for the X-ray diffraction are obtained by slow evaporation of a solution of the title compound in DMF at room temperature.

### 3. Refinement

The H atoms were positioned geometrically (N—H = 0.86 Å, C—H = 0.93 Å, O—H = 0.82 Å) and refined as riding on their carriers with *U*_iso_(H)= 1.2*U*_eq_(C,N,O).

### Crystal data

**Table:**

C_12_H_8_BrClN_2_O_3_	*F*(000) = 680
*M**_r_* = 343.56	*D*_x_ = 1.733 Mg m^−^^3^
Monoclinic, *P*2_1_/*c*	Mo *K*α radiation, λ = 0.71073 Å
Hall symbol: -P 2ybc	Cell parameters from 4049 reflections
*a* = 16.7237 (9) Å	θ = 2.8–24.5°
*b* = 7.7455 (4) Å	µ = 3.33 mm^−^^1^
*c* = 10.1868 (5) Å	*T* = 293 K
β = 93.557 (2)°	Block, yellow
*V* = 1316.99 (12) Å^3^	0.35 × 0.30 × 0.25 mm
*Z* = 4	

### Data collection

**Table:**

Bruker AAPEXII CCD Diffractometer	2996 independent reflections
Radiation source: fine-focus sealed tube	2029 reflections with *I* > 2σ(*I*)
Graphite monochromator	*R*_int_ = 0.032
ω and φ scan	θ_max_ = 27.4°, θ_min_ = 2.4°
Absorption correction: multi-scan (*SADABS*; Bruker, 2004)	*h* = −21→21
*T*_min_ = 0.324, *T*_max_ = 0.435	*k* = −10→8
13728 measured reflections	*l* = −13→13

### Refinement

**Table:**

Refinement on *F*^2^	Primary atom site location: structure-invariant direct methods
Least-squares matrix: full	Secondary atom site location: difference Fourier map
*R*[*F*^2^ > 2σ(*F*^2^)] = 0.034	Hydrogen site location: inferred from neighbouring sites
*wR*(*F*^2^) = 0.085	H-atom parameters constrained
*S* = 1.02	*w* = 1/[σ^2^(*F*_o_^2^) + (0.0346*P*)^2^ + 0.5776*P*] where *P* = (*F*_o_^2^ + 2*F*_c_^2^)/3
2996 reflections	(Δ/σ)_max_ = 0.001
172 parameters	Δρ_max_ = 0.39 e Å^−^^3^
0 restraints	Δρ_min_ = −0.34 e Å^−^^3^

### Special details

**Table:**

Geometry. All e.s.d.'s (except the e.s.d. in the dihedral angle between two l.s. planes) are estimated using the full covariance matrix. The cell e.s.d.'s are taken into account individually in the estimation of e.s.d.'s in distances, angles and torsion angles; correlations between e.s.d.'s in cell parameters are only used when they are defined by crystal symmetry. An approximate (isotropic) treatment of cell e.s.d.'s is used for estimating e.s.d.'s involving l.s. planes.
Refinement. Refinement of *F*^2^ against ALL reflections. The weighted *R*-factor *wR* and goodness of fit *S* are based on *F*^2^, conventional *R*-factors *R* are based on *F*, with *F* set to zero for negative *F*^2^. The threshold expression of *F*^2^ > σ(*F*^2^) is used only for calculating *R*-factors(gt) *etc*. and is not relevant to the choice of reflections for refinement. *R*-factors based on *F*^2^ are statistically about twice as large as those based on *F*, and *R*- factors based on ALL data will be even larger.

### Fractional atomic coordinates and isotropic or equivalent isotropic displacement parameters (Å^2^)

**Table:**

	*x*	*y*	*z*	*U*_iso_*/*U*_eq_	
C1	0.30900 (18)	0.0948 (4)	0.5312 (4)	0.0704 (9)	
H1	0.2756	0.0562	0.5947	0.084*	
C2	0.28824 (18)	0.1059 (4)	0.4065 (3)	0.0678 (8)	
H2	0.2389	0.0761	0.3657	0.081*	
C3	0.35486 (17)	0.1718 (4)	0.3450 (2)	0.0558 (7)	
H3	0.3579	0.1954	0.2560	0.067*	
C4	0.41272 (14)	0.1937 (3)	0.4394 (2)	0.0386 (5)	
C5	0.49358 (14)	0.2608 (3)	0.4308 (2)	0.0388 (5)	
C6	0.66294 (15)	0.3109 (3)	0.6435 (2)	0.0410 (6)	
H6	0.6461	0.2574	0.7187	0.049*	
C7	0.74492 (14)	0.3741 (3)	0.6395 (2)	0.0377 (5)	
C8	0.76987 (14)	0.4658 (3)	0.5306 (2)	0.0383 (5)	
C9	0.84795 (15)	0.5274 (3)	0.5343 (2)	0.0467 (6)	
C10	0.90118 (15)	0.4977 (3)	0.6409 (3)	0.0516 (7)	
H10	0.9531	0.5410	0.6421	0.062*	
C11	0.87656 (16)	0.4041 (3)	0.7446 (2)	0.0484 (6)	
C12	0.79961 (15)	0.3424 (3)	0.7459 (2)	0.0458 (6)	
H12	0.7839	0.2796	0.8178	0.055*	
N1	0.53833 (11)	0.2682 (3)	0.54589 (17)	0.0418 (5)	
H1A	0.5194	0.2354	0.6184	0.050*	
N2	0.61464 (12)	0.3296 (2)	0.54270 (18)	0.0410 (5)	
O1	0.38640 (12)	0.1475 (3)	0.55681 (17)	0.0626 (5)	
O2	0.51905 (11)	0.3068 (3)	0.32640 (15)	0.0575 (5)	
O3	0.72099 (10)	0.4955 (2)	0.42286 (14)	0.0485 (4)	
H3A	0.6769	0.4528	0.4330	0.073*	
Cl1	0.94376 (5)	0.36440 (11)	0.87885 (8)	0.0748 (2)	
Br1	0.881917 (19)	0.65331 (5)	0.38959 (3)	0.07798 (15)	

### Atomic displacement parameters (Å^2^)

**Table:**

	*U*^11^	*U*^22^	*U*^33^	*U*^12^	*U*^13^	*U*^23^
C1	0.0489 (18)	0.075 (2)	0.090 (2)	−0.0171 (16)	0.0222 (16)	0.0091 (18)
C2	0.0467 (18)	0.069 (2)	0.086 (2)	−0.0055 (15)	−0.0126 (16)	−0.0198 (17)
C3	0.0533 (17)	0.081 (2)	0.0322 (12)	0.0000 (15)	−0.0055 (11)	−0.0015 (12)
C4	0.0414 (14)	0.0433 (14)	0.0314 (11)	−0.0011 (11)	0.0038 (10)	−0.0031 (10)
C5	0.0415 (14)	0.0453 (14)	0.0299 (11)	0.0020 (11)	0.0034 (10)	−0.0031 (10)
C6	0.0397 (14)	0.0484 (15)	0.0350 (12)	0.0023 (11)	0.0024 (10)	0.0026 (10)
C7	0.0378 (13)	0.0383 (13)	0.0367 (12)	0.0038 (10)	−0.0010 (9)	−0.0033 (10)
C8	0.0379 (13)	0.0399 (14)	0.0369 (11)	0.0030 (11)	−0.0006 (10)	−0.0007 (10)
C9	0.0444 (15)	0.0466 (15)	0.0493 (13)	0.0000 (12)	0.0038 (11)	0.0031 (11)
C10	0.0383 (15)	0.0494 (16)	0.0663 (17)	−0.0004 (12)	−0.0044 (12)	−0.0011 (13)
C11	0.0464 (15)	0.0478 (15)	0.0487 (14)	0.0079 (12)	−0.0145 (11)	−0.0041 (12)
C12	0.0480 (15)	0.0473 (15)	0.0411 (13)	0.0043 (12)	−0.0045 (11)	0.0035 (11)
N1	0.0348 (11)	0.0605 (13)	0.0302 (9)	−0.0051 (10)	0.0035 (8)	0.0000 (9)
N2	0.0353 (11)	0.0491 (12)	0.0385 (10)	−0.0011 (9)	0.0028 (8)	−0.0032 (9)
O1	0.0529 (12)	0.0929 (15)	0.0428 (10)	−0.0065 (11)	0.0091 (8)	0.0075 (9)
O2	0.0502 (11)	0.0906 (14)	0.0323 (9)	−0.0104 (10)	0.0073 (8)	0.0032 (9)
O3	0.0406 (10)	0.0673 (12)	0.0368 (8)	−0.0025 (8)	−0.0027 (7)	0.0072 (8)
Cl1	0.0626 (5)	0.0837 (6)	0.0734 (5)	0.0051 (4)	−0.0337 (4)	0.0057 (4)
Br1	0.0531 (2)	0.1004 (3)	0.0810 (2)	−0.01171 (17)	0.00947 (15)	0.03418 (18)

### Geometric parameters (Å, º)

**Table:**

C1—C2	1.299 (4)	C7—C12	1.396 (3)
C1—O1	1.367 (4)	C7—C8	1.403 (3)
C1—H1	0.9300	C8—O3	1.347 (3)
C2—C3	1.407 (4)	C8—C9	1.388 (3)
C2—H2	0.9300	C9—C10	1.380 (3)
C3—C4	1.332 (3)	C9—Br1	1.885 (2)
C3—H3	0.9300	C10—C11	1.366 (4)
C4—O1	1.348 (3)	C10—H10	0.9300
C4—C5	1.456 (3)	C11—C12	1.373 (4)
C5—O2	1.224 (3)	C11—Cl1	1.743 (2)
C5—N1	1.352 (3)	C12—H12	0.9300
C6—N2	1.275 (3)	N1—N2	1.364 (3)
C6—C7	1.459 (3)	N1—H1A	0.8600
C6—H6	0.9300	O3—H3A	0.8200
			
C2—C1—O1	111.1 (3)	O3—C8—C9	119.1 (2)
C2—C1—H1	124.5	O3—C8—C7	122.4 (2)
O1—C1—H1	124.5	C9—C8—C7	118.5 (2)
C1—C2—C3	106.7 (3)	C10—C9—C8	121.6 (2)
C1—C2—H2	126.6	C10—C9—Br1	119.4 (2)
C3—C2—H2	126.6	C8—C9—Br1	119.00 (18)
C4—C3—C2	106.6 (2)	C11—C10—C9	119.1 (2)
C4—C3—H3	126.7	C11—C10—H10	120.5
C2—C3—H3	126.7	C9—C10—H10	120.5
C3—C4—O1	110.1 (2)	C10—C11—C12	121.3 (2)
C3—C4—C5	129.7 (2)	C10—C11—Cl1	119.3 (2)
O1—C4—C5	120.2 (2)	C12—C11—Cl1	119.3 (2)
O2—C5—N1	122.5 (2)	C11—C12—C7	120.0 (2)
O2—C5—C4	122.0 (2)	C11—C12—H12	120.0
N1—C5—C4	115.4 (2)	C7—C12—H12	120.0
N2—C6—C7	119.3 (2)	C5—N1—N2	117.55 (19)
N2—C6—H6	120.4	C5—N1—H1A	121.2
C7—C6—H6	120.4	N2—N1—H1A	121.2
C12—C7—C8	119.4 (2)	C6—N2—N1	119.2 (2)
C12—C7—C6	119.4 (2)	C4—O1—C1	105.5 (2)
C8—C7—C6	121.2 (2)	C8—O3—H3A	109.5
			
O1—C1—C2—C3	−0.8 (4)	C7—C8—C9—Br1	−179.23 (17)
C1—C2—C3—C4	0.9 (4)	C8—C9—C10—C11	0.7 (4)
C2—C3—C4—O1	−0.7 (3)	Br1—C9—C10—C11	−179.00 (19)
C2—C3—C4—C5	−178.9 (3)	C9—C10—C11—C12	−1.5 (4)
C3—C4—C5—O2	−0.6 (4)	C9—C10—C11—Cl1	179.38 (19)
O1—C4—C5—O2	−178.7 (2)	C10—C11—C12—C7	0.4 (4)
C3—C4—C5—N1	179.5 (3)	Cl1—C11—C12—C7	179.61 (18)
O1—C4—C5—N1	1.4 (3)	C8—C7—C12—C11	1.4 (4)
N2—C6—C7—C12	−175.3 (2)	C6—C7—C12—C11	−178.7 (2)
N2—C6—C7—C8	4.6 (3)	O2—C5—N1—N2	−0.1 (4)
C12—C7—C8—O3	177.6 (2)	C4—C5—N1—N2	179.77 (19)
C6—C7—C8—O3	−2.3 (3)	C7—C6—N2—N1	179.1 (2)
C12—C7—C8—C9	−2.1 (3)	C5—N1—N2—C6	−167.9 (2)
C6—C7—C8—C9	178.0 (2)	C3—C4—O1—C1	0.2 (3)
O3—C8—C9—C10	−178.6 (2)	C5—C4—O1—C1	178.6 (2)
C7—C8—C9—C10	1.1 (4)	C2—C1—O1—C4	0.4 (4)
O3—C8—C9—Br1	1.1 (3)		

### Hydrogen-bond geometry (Å, º)

**Table:**

*D*—H···*A*	*D*—H	H···*A*	*D*···*A*	*D*—H···*A*
N1—H1*A*···O2^i^	0.86	2.14	2.953 (2)	157
C3—H3···O1^ii^	0.93	2.44	3.324 (3)	159
C6—H6···O2^i^	0.93	2.50	3.263 (3)	139
O3—H3*A*···N2	0.82	1.84	2.564 (3)	146

Symmetry codes: (i) *x*, −*y*+1/2, *z*+1/2; (ii) *x*, −*y*+1/2, *z*−1/2.
